# Strengthening parenting in conflict-affected communities: development of the Caregiver Support Intervention

**DOI:** 10.1017/gmh.2020.8

**Published:** 2020-06-02

**Authors:** Kenneth E. Miller, Heba Ghalayini, Maguy Arnous, Fadila Tossyeh, Alexandra Chen, Myrthe van den Broek, Gabriela V. Koppenol-Gonzalez, Joy Saade, Mark J.D. Jordans

**Affiliations:** 1 War Child Holland, Amsterdam, The Netherlands; 2 War Child Holland, Gaza, Palestine; 3 War Child Holland, Beirut, Lebanon; 4 War Child Holland, Tripoli, Lebanon; 5 Harvard University, Cambridge, USA; 6 Amsterdam Institute of Social Science Research, University of Amsterdam, Amsterdam, The Netherlands

**Keywords:** parenting, refugees, psychosocial, armed conflict, children

## Abstract

**Background:**

There is robust evidence that compromised parenting, stemming from persistently high stress, mediates the impact of war and displacement on children's mental health and psychosocial wellbeing. Parenting interventions generally prioritize the acquisition of parenting knowledge and skills, while under-attending to parental stress and distress. This paper describes the development of the Caregiver Support Intervention (CSI), a nine-session group intervention for conflict-affected parents of children aged 3–13, that aims to strengthen parenting both indirectly, by lowering stress and improving psychosocial wellbeing among parents, and directly, by increasing knowledge and skill related to positive parenting.

**Methods:**

We describe the multi-phase, iterative process by which we developed the CSI, and illustrate the essential role of community input in shaping the intervention and strengthening its cultural fit and perceived usefulness. We used focus group data from participants in successive cycles of implementation, feedback, and revision, as well as quantitative data and expert consultation to develop a culturally and empirically grounded intervention.

**Results:**

This mixed-method, iterative approach to intervention development enabled us to develop a psychosocial intervention for conflict-affected caregivers that is feasible, acceptable, and perceived by participants as useful in addressing their own wellbeing and their parenting. Focus group data support the underlying model in which caregiver wellbeing powerfully influences parenting.

**Conclusions:**

Programs aimed at strengthening parenting in conflict-affected communities should substantively address caregiver wellbeing. An iterative approach incorporating community feedback can help ensure intervention acceptability and feasibility. We also illustrate the feasibility of involving men in parenting interventions.

There is compelling evidence that compromised parenting mediates the impact of war and displacement on children's mental health (Palosaari *et al*., 2013; Panter-Brick *et al*., 2014*b*; Saile *et al*., 2014; Song *et al*., 2014; El-Khani *et al*., 2016; Sim *et al*., 2018; Eltanamly *et al*., 2019; Scharpf *et al*., 2019). War-related violence and loss, coupled with continuous exposure to stressors such as poverty, unemployment, poor housing, a lack of access to basic resources, and the loss of social support networks, exact a toll on parents' mental health and psychosocial wellbeing (Eltanamly *et al*., 2019). As parental stress and distress increase, parents may become more likely to engage in harsh parenting, and less likely to engage in the kinds of supportive, nurturing interactions that contribute to healthy child development (Biglan *et al*., 2012; Jackson and Choi, 2018), and that might buffer children from the chaos and deprivation of conflict and refugee settings (Masten and Narayan, 2012; Betancourt *et al*., 2013).^1^

In a recent systematic review and meta-analysis, Eltanamly *et al.* found that parental stress and distress partially mediate the impact of armed conflict on children via two primary pathways: a reduction in parental warmth and an increase in harsh parenting (Eltanamly *et al*., 2019). In their study of Syrian parents in Lebanon, for example, Sim *et al*. (2018) found that parental stress and distress stemming from the hardships of displacement were linked to a greater use of harsh discipline and a reduction in parental supervision and positive parent–child interactions. In Afghanistan, prolonged armed conflict has contributed to widespread unemployment and poverty, which have led to increased parental stress and a heightened risk of family violence, including violence toward children (Eggerman and Panter-Brick, 2010; Panter-Brick *et al*., 2014*b*). Research with Palestinian families found that parental stress and distress fully mediated the impact of political violence on children's mental health (Khamis, 2016), and that parental stress related to economic deprivation predicted child maltreatment. In East Timor, men's exposure to political violence contributed to an increase in violence against their wives, which in turn led an increase among mothers in the use of violence against their children (Rees *et al*., 2015). The latter finding is consistent with other studies that have found that intimate partner violence increases in war-affected communities, exposing children to frightening violence and undermining the parenting of their abused and distressed mothers (Clark *et al*., 2010; Eggerman and Panter-Brick, 2010; Wachter *et al*., 2018).

The adverse impact of adversity on parenting among refugees is by no means absolute; many conflict-affected parents manage to nurture and protect their children despite their experience of violence, loss, and ongoing adversity (Betancourt *et al*., 2013; Song *et al*., 2014; Alleyne-Green *et al*., 2019). Nonetheless, the finding of compromised parenting stemming from the combination of war exposure and daily stressors is robust, and has led to an interest in strengthening parenting in refugee and other conflict-affected families (Jordans *et al*., 2013; Puffer *et al*., 2015, 2017; Singla *et al*., 2015).

The theoretical underpinning of evidence-based group parenting interventions varies; typically, however, they have a strong behavioral focus grounded in social learning theory (Sanders *et al*., 2014). They generally also share the proximal aims of reducing harsh parenting and fostering positive parent–child interactions, reflecting the importance of a safe and nurturing environment for children's social and emotional development (Biglan *et al*., 2012). Implicit in their emphasis on parent training is a theory of change that explains sub-optimal parenting primarily as a lack of essential parenting knowledge and skills, which are taught through a combination of didactic and interactive group activities.

With some notable exceptions (Brown *et al*., 2013; Coatsworth *et al*., 2015), there has been little substantive attention to parents' own psychosocial wellbeing in parenting interventions for families living in adversity. This is surprising, given the robust evidence that chronic adversity undermines parenting (McLoyd, 1990; Masarik and Conger, 2017), including among refugee parents (Sim *et al*., 2018; Eltanamly *et al*., 2019). The linkages among adversity, parental wellbeing, and parenting are captured in Conger's Family Stress Model (FSM) (Conger *et al*., 2010). The model posits a pathway beginning with exposure to persistent adversity, leading to heightened parental stress, which in turn adversely impacts parenting, eventually compromising children's mental health and ongoing development. The FSM has found empirical support in studies of families experiencing diverse forms of chronic stress (Masarik and Conger, 2017), including, most recently, Syrian refugees in Lebanon (Sim *et al*., 2018). The FSM model would suggest that at least some of the sub-optimal parenting seen among refugees is due not to a deficit in knowledge and skills, but to the impact of persistently high stress on parents' ability to make use of the knowledge and abilities they already possess.

Parent training programs in conflict-affected communities *have* shown moderate effects on parenting outcomes in several studies (e.g. Puffer *et al*. 2015, 2017), and on child mental health (Pedersen *et al*., 2019), suggesting that parenting can be strengthened, even in highly stressed families, without substantively targeting parents' own wellbeing. Raising children is challenging, and undoubtedly many parents can benefit from the additional knowledge and skills taught in parent training programs. Cultural norms (e.g. that sanction harsh parenting), as well as parents' experience with their own parents, clearly influence parenting behavior and may be effectively addressed through knowledge and skills-based training programs. Although there is a paucity of research on the specific mechanisms by which parenting interventions achieve their effects, a study by Mejia *et al.* in a low-resource area of Panama (Mejia *et al*., 2016) found that increased knowledge and skill in positive parenting methods did contribute to changes in parenting.

Interestingly, the study by Mejia *et al*. (2016) also found that increased emotion regulation among parents also improved parenting, suggesting that improved psychosocial wellbeing may be a collateral benefit of parenting interventions which in turn accounts for changes in parenting behavior. This finding is consistent with a review of the collateral benefits of parenting interventions for parents' own wellbeing, which found modest improvements on various mental health outcomes (Bennett *et al*., 2013).

Given the well-documented relationship between parental wellbeing and parenting, we might ask whether intervention effects could be strengthened by focusing more intentionally and substantively on reducing stress and increasing psychosocial wellbeing among parents, particularly among families living highly stressful conditions. If we consider the impact of adversity on parental wellbeing and subsequently on parenting, our model becomes a model of parenting *compromised* by the experience of both acute and continuous stress. This model, depicted in Fig. 1, suggests a theory of change in which lowered stress and improved psychosocial wellbeing enable parents to make better use of the knowledge and skills they already possess, as well as new concepts and methods acquired through parent training.

**Fig. 1.**
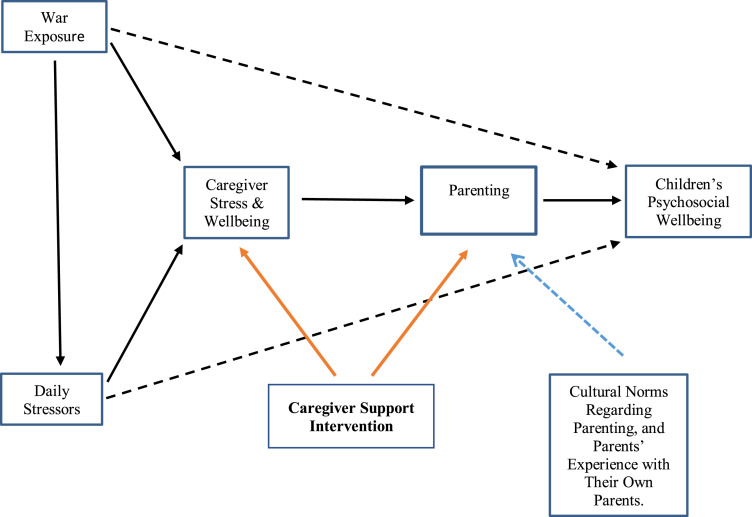
Conceptual model underlying the CSI. Dotted black lines indicate direct effects of war exposure and daily stressors on children's psychosocial wellbeing. Solid black lines indicate pathways from armed conflict to parenting, mediated by caregiver stress and wellbeing. Dotted blue line indicates the influence of cultural norms on parenting, as well as parents' experience with their own parents. Solid orange lines indicate the dual focus of CSI on targeting parenting indirectly via impact on caregiver stress and wellbeing, and directly through focus on positive parenting.

Guided by the model in Fig. 1, War Child Holland (WCH, a Netherlands-based non-governmental organization that addresses the psychosocial, educational, and child protection needs of children and families affected by armed conflict) developed the Caregiver Support Intervention (CSI). The CSI is a nine-session group intervention for parents and other primary caregivers of children aged 3–12 affected by armed conflict and forced migration. The CSI aims to strengthen parenting through two pathways: indirectly, by lowering stress and improving psychosocial wellbeing among parents, and directly, by increasing knowledge and skill related to positive parenting (i.e. parenting that is nurturing, consistent, authoritative, and non-violent). The CSI is implemented by trained and supervised non-specialists, and targets all parents, regardless of the level of distress and parenting difficulties. Weekly sessions last for two hours, and combine interactive and didactic activities.

Our primary aim in this paper is to describe the iterative process by which we developed the CSI. We also discuss the methods we have employed to successfully recruit and retain men in the CSI, in a cultural context where men are widely perceived as uninterested in parent-focused interventions. We begin by first briefly presenting the key elements of the CSI, as well as the rationale for their inclusion.

## Part one: the CSI curriculum

Guided by the model in Fig. 1, we felt the priority in the first part of the CSI should be on fostering parental wellbeing. Thus, the focus of the first four sessions is on lowering stress and strengthening wellbeing through the social support of the group, and by enhancing the capacity to cope with stress, frustration, and anger. The emphasis on stress management continues throughout the intervention; however, the primary focus in the second half of the intervention is on parenting in adversity, and draws substantially from the literature on positive parenting (Pastor *et al*., 2015). Throughout the intervention, one or more stress management techniques are introduced in each session, and participants are encouraged to practice the techniques several times each week. Recordings of all stress management activities are provided to participants for use on their phones or on mp3 players. Table 1 lists the main topic and stress management activities for each session.

**Table 1.** CSI sessions, modules, and stress management/relaxation methods

Session	Topic	Module
1	Introduction and Group Building *SM*^a^*: Participants' own methods of coping with stress*	Caregiver Wellbeing
2	Stress and Relaxation *SM: Counting the Breath*	Caregiver Wellbeing
3	Lowering our Stress *SM: Stepping Back from our Thoughts, Grounding*	Caregiver Wellbeing
4	Coping with Frustration and Anger *SM: Peaceful Walking, various anger management techniques*	Caregiver Wellbeing
5	Parental Stress and Influence *SM: Stepping Back from our Thoughts (repeat)*	Parenting in Adversity
6	Increasing our Influence as Parents, Part I: Positive Attention *SM: Guided Visualization: A Safe Place*	Parenting in Adversity
7	Increasing our Influence as Parents, Part 2: Effective Discipline *SM: Informal Breathing Practice*	Parenting in Adversity
8	Positive Parenting: Practice *SM: Participants Choose Any Stress Management/Relaxation Method*	Parenting in Adversity
9	Looking Back, Looking Forward	Closure

a SM, stress management/relaxation technique taught during the session.

The structure of the sessions follows a consistent format. Following a fun energizer, the first 25 min of each session are spent reviewing the previous week's home practice of the stress management activities; the group collectively problem-solves any barriers to practice that anyone has experienced. The main theme of the session is then addressed using a combination of participatory and didactic methods. The sessions end with the introduction and practice of a new stress management technique, and a brief discussion of any barriers that might arise to home practice during the coming week. The heavily participatory nature of the sessions is meant to make the experience engaging and empowering, and to foster a sense of social support of group members.

In selecting content for the CSI, we adopted a culturally integrative approach, drawing on well-researched concepts and methods from diverse cultural contexts. For example, in the sessions on managing and reducing stress, we explore ways of stepping back from ‘thinking too much’ (*Am bfakr kteer),* which entails thinking constantly about one's problems, and worrying persistently about the future. *Am bfakr kteer* is salient throughout the Middle East and Central Asia (Miller *et al*., 2006; Kaiser *et al*., 2015). It has strong parallels with the Buddhist emphasis on *overthinking* as a source of suffering (Kornfield, 1993) and with the western concept of rumination, common to both depression and anxiety. We also address anger and frustration using the Arabic and Turkish concept of *Asabi*. To become *Asabi* literally means to ‘lose one's nerve’, but the term refers to becoming aggressively irritable or angry in response to life stress (Miller *et al*., 2006). We selected these cultural idioms because they are salient in the communities where the CSI was being developed. Our identification of locally salient stress reactions and expressions of distress was informed by the extensive clinical and research experience of our team in the region, as well as a review of the relevant literature. At the same time, we sought to identify expressions of stress and distress that reflect psychological states and processes found transculturally (i.e. stress-related irritability, overthinking/rumination), an important factor in making the intervention readily adaptable to other cultural contexts.

The stress management techniques used in the CSI draw on mindfulness practices which originated in South and Southeast Asia, but which have been widely adapted for use in other cultural contexts (Hayes, 2002; Fung, 2015; Tol *et al*., 2018). The various practices share a focus on helping participants recognize and disengage from stress-inducing thoughts, either by re-focusing their attention on their breath or on another activity such as walking, or by using calming visual imagery. We also incorporated simple relaxation exercises such as progressive muscle relaxation, as well as anger management techniques such as counting to 10 or taking a short walk, before reacting to an emotionally charged situation. To avoid religious connotations that might cause discomfort in the traditional Muslim communities where the CSI was developed, we avoided the term *mindfulness*, with its Buddhist roots, instead referring to all of the stress management activities simply as ‘relaxation exercises.’

In the parenting sessions, we emphasize concepts and methods that have been found to support healthy psychosocial development in children across a wide diversity of cultures (Biglan *et al*., 2012; Knerr *et al*., 2013; Sanders *et al*., 2014; Ward *et al*., 2016). After first exploring the ways in which stress can affect parenting, we then focus on increasing positive attention and fostering nurturing parent–child interactions. We address the adverse impact of harsh parenting on children's mental and physical wellbeing, and explore the use of authoritative, non-violent approaches to behavior management.

Once a zero draft protocol was developed, it was circulated for review among colleagues in Gaza and Lebanon, where the intervention would subsequently be implemented, and among a group of experts in parenting interventions. Feedback was incorporated, and we then moved to the next phase, the use of an iterative process that was key to developing a culturally acceptable and feasible intervention.

## Part two: an iterative approach to developing the CSI

**Fig. 2.**
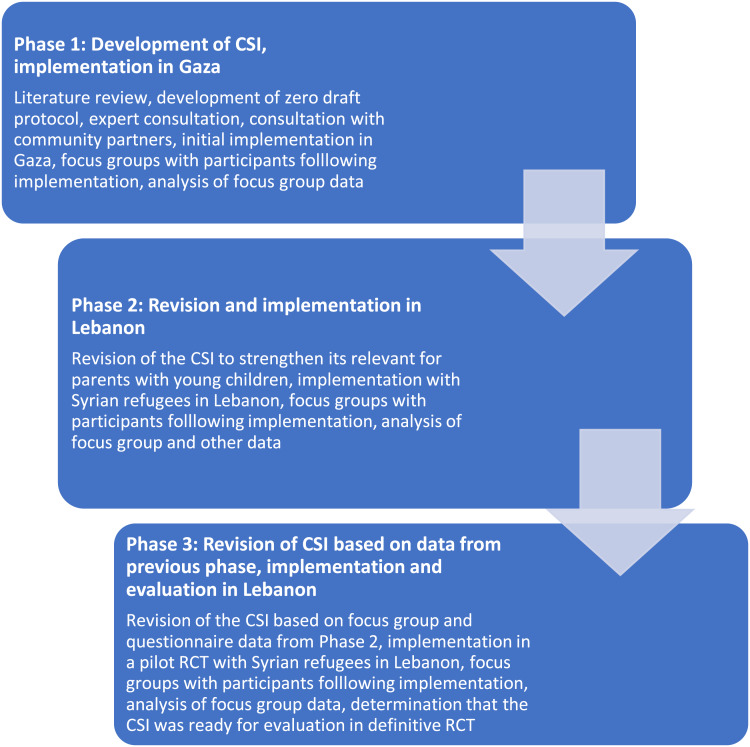
Multiphase development of the CSI.

The development of the CSI followed a multiphase, iterative process, illustrated in Fig. 2. This process was similar to the person-centered approach described by Yardley *et al*. (2015), in which in-depth and repeated participant feedback plays a critical role in the development of new interventions.

### Phase 1: development of initial curriculum, implementation and practice run in Gaza

In Phase 1, we first reviewed the relevant literature (e.g. parenting programs, mindfulness interventions, stress management techniques, regionally salient idioms and indicators of distress), and consulted with experts in each of these areas. An eight-session curriculum was drafted, aimed at parents of children aged 10–15.

The intervention was initially implemented in Gaza, where the staff of WCH's Gaza office had observed high levels of stress among parents. Poverty is endemic in Gaza, unemployment exceeds 40%, water and electricity are scarce, medical care is limited, and the deprivations of everyday life are punctuated by intermittent shelling from Israel (New York Times, 2010; UN News, 2017). Our primary aims in this initial implementation were to assess the cultural fit and perceived usefulness of the CSI, and to see whether we could successfully recruit and retain men in the intervention – a persistent challenge in parenting interventions (Panter-Brick *et al*., 2014*a*). We recruited three groups of women and three groups of men, all of whom had at least one child between the ages of 10 and 15. The women and men were not related to each other in this first study. The groups had between 10 and 12 participants, with a mean age of 38 and a broad age range of 23–70. The mean number of children per participant was 4.5. The groups were conducted in the offices of community-based organizations with which WCH collaborates.

Focus groups were conducted with the participants from each of the CSI groups during the 2 weeks following the last session. Forty-eight of the 72 participants attended the focus groups, and were evenly split among women and men. The groups lasted between one and a half to two hours, and were facilitated by WCH staff members trained in focus group facilitation. Participants who could not attend focus groups, primarily due to illness, work, or other scheduling conflicts, were contacted by phone and interviewed individually, to ensure their views were included. Because their responses did not differ meaningfully from those in the focus groups, we limit our discussion here to the focus group data.

We developed a focus group discussion guide to help us learn about participants' reactions to all aspects of the CSI, from program content, to quality of implementation, to logistics such as transportation, setting, and the duration and number of sessions. Data were recorded by hand, translated into English, and entered into the qualitative software program DeDoose. Note takers were trained to take verbatim notes. A code book was developed that reflected the main topics in the discussion guide, with additional codes added as needed. To achieve inter-rater agreement, we used consensus coding (Bradley *et al*., 2007). Pairs of coders independently coded each focus group transcript, then met to compare their coding and resolve any discrepancies. Data were analyzed using thematic analysis (Nowell *et al*., 2017).

#### Key findings

*Engagement with the CSI was high.* There were no drop-outs, and 85% of participants completed seven or all eight of the sessions (89% of men and 80% of women).

*The stress management exercises were highly valued, and many participants reported practicing them three or more times per week.* People reported using the techniques to relax and to fall asleep more easily. People linked their use of the exercises to feeling calmer and better able to respond to their children in more supportive, nurturing ways. One man commented:

‘I found that the relaxation techniques soothe me and help in taking me away from my sorrows for a while. I also became more tender and empathetic to my children and I started to be more careful and sensitive about my attitudes when I deal with them.’

Several participants described teaching the activities to their spouses, children, and siblings, as illustrated by this woman's response:

‘The relaxation exercises were very useful, soothing, and relaxing. I also started to do the relaxation exercises and the fun activities that we implemented during the sessions with my children at home.’

*Participants described a reduction in harsh parenting and an increase in parental warmth*. Participants attributed this partly to the sessions on positive parenting, which included a discussion of the adverse effects of harsh parenting. One father's comment is illustrative:

‘I used to hit and beat my children before if they did anything wrong. But after learning how much harm hitting or slapping kids can cause, I started to change and I started to ask my wife to stop beating them. I convinced her that beating is not the right way to regulate your children.’

Shifting away from harsh parenting toward a greater use of positive discipline methods was facilitated by parents becoming better able to first calm themselves before responding to their children's problem behavior. The stress management techniques helped parents feel calmer and more rested, while the anger management techniques provided immediately accessible strategies for lowering their reactivity in moments of conflict. Under conditions of lower arousal, parents felt better able to respond constructively. This in turn led to feelings of greater parental efficacy and more harmonious parent–child relationships. One woman described this process with regard to her daughter's schoolwork:

‘It helped me to control my anger. My daughter got a low result in a math exam, and I used to be angry with her, but I did something different this time. I told her that it's ok and that I will help her to get better marks. I started to be more encouraging and reinforcing with her and her grades became higher as she got 19/20 in her last exam.’

A male participant described a similar shift that stemmed from his increased ability to manage his anger:

‘The sessions made a difference in the way I deal with my children. In the past I used to be angry with them when they made trouble, but after the sessions I started to gain more control over my reactions and emotions.’

Several participants linked the stress management activities to improved sleep, which left them more rested and emotionally available for their children. One mother said, ‘I started to sleep better because of the relaxation exercises and my relationship with my children became stronger and warmer.’

### Phase 2: revision, implementation, and practice run with Syrian refugees in Lebanon

Shortly after completing our evaluation of the CSI in Gaza, we went on to further develop and evaluate the CSI with Syrian refugees in Lebanon. Lebanon, with a population of 6.8 million, currently hosts nearly 1.5 million Syrians, refugees of a prolonged and devastating war that has displaced nearly 12 million people, including 6.2 million within Syria and 5.6 million in neighboring countries and beyond (Yassin, 2018). Numerous studies and field reports have documented high levels of stress among Syrian refugee parents, the great majority of whom are raising their children in conditions of poverty, uncertainty regarding the future, and a welcome that has grown increasingly thin as the war has dragged on (Sim *et al*., 2018; VASyR., 2018).

Prior to conducting a small-scale ‘practice run’ of the CSI in Lebanon, we first set about revising the intervention based on several considerations. First, given the emphasis on stress management and strengthening parental wellbeing, we felt that the CSI should be helpful to parents of a much broader age range than early adolescence. In consultation with several early childhood and parenting experts, we revised the parenting sessions to strengthen their relevance for parents of children in early and middle childhood. This added an additional session to the intervention, resulting in the current nine-session program. Second, we re-recorded the stress management exercises in formal Arabic, in response to feedback from Syrian community members that they felt uncomfortable with Gazan Arabic. Third, we developed a manual for the training of CSI facilitators, created a fidelity checklist to be completed by all facilitators after each session, as well as a competency checklist to be used by trainers to assess progress during the training of new facilitators, and by the supervisor during on-site observation and coaching of facilitators.

We then implemented the revised CSI with a sample of 74 Syrian refugee families in North Lebanon: three groups of women and three groups of men. The groups had 12–13 people each, with a mean age of 38.6 (range 19–63) and a mean of 3.7 children per family. Our aims, as in Gaza, were to assess the cultural fit and perceived usefulness of the revised intervention, and to evaluate the feasibility of recruiting and retaining men in the program. We did not recruit couples for this practice run of the revised intervention, aiming instead only to recruit the target number of women and men (i.e. male and female participants were not spouses or partners of each other).

Focus group discussions were conducted with each of the CSI groups during the 2 weeks following the last session. In total, 66% of CSI participants participated in the focus groups (25 women and 24 men). We again trained the note takers to take verbatim notes, this time using pairs of note takers who relieved each other every 20–30 min. Focus group data from the practice cycle were coded and analyzed using the qualitative data software program NVivo v.12, and followed the same process as that described for the Gaza data. The groups lasted for about 2 h, and were facilitated by the research coordinator, a Lebanese woman trained in focus group facilitation.

#### Key findings

Our findings differed from those in the Gaza implementation in several interesting ways. Attendance in the intervention was again high, with 75% of women and 73% of men attending at least seven of the nine sessions. In contrast to Gaza, however, where we had no drop-outs, 11 of the 38 men in Lebanon dropped out of the intervention, as did nine of the 36 women. To some extent, the lower completion and higher dropout rates reflect differences in the two contexts: there was considerably more sickness among the Syrian sample, we experienced transportation difficulties in Tripoli, participants were sometimes busy with visits to or from Syria, and the sessions occasionally conflicted with income generation opportunities for men.

However, these factors do not entirely explain the different completion and drop-out rates between the two settings. In focus groups conducted with participants in Tripoli, including those who did not complete the intervention, we gained valuable insights into several aspects of the intervention that needed to be modified.

*Reluctance among men to try the stress management exercises*. While women were enthusiastic about all aspects of the CSI, a majority of men expressed wariness regarding the stress management exercises. Although several men did practice them regularly and found them beneficial to their wellbeing and their parenting, a majority stated that they had their own ways of coping with stress, and wanted to see the science behind the CSI stress management methods before they would be willing to try them. Interestingly, this resistance was not evident with the frustration and anger management techniques, which men viewed quite positively. They gave numerous examples of using methods such as counting to 10 or taking a short walk before responding to a child's misbehavior, which then allowed them to respond in constructive, non-violent ways.

In contrast, women consistently reported using and benefiting from the stress management exercises, and linked their practice of them to increased wellbeing, a shift toward positive parenting and away from harsh parenting, and to warmer, more positive relationships with their children and spouses. Consider the contrast in these representative responses from women and men.

First, comments from two women:

‘The relaxation exercises helped me manage my reaction to things, slowed my thinking and gave me comfort. Comfort to me alone. It gave me emotional and physical comfort at the same time. It was a boost forward for me.’

‘The counting method and taking the other's perspective helped me with my children and husband. Now I am able to be in control and my husband's behavior also changed. When he used to get angry, he refused to talk about his perspective. But now when I count to 10, I opened the space for him to relax and start talking in a calmer manner.’

And from men:

‘I don't really need the exercises, I can just go out of the house. I pay more attention to myself and my health. I go see a friend.’

‘Personally, I go for a walk when I'm angry, and I feel better.’

‘I would have liked more scientific research.’

*Perceived irrelevance of the early childhood development content for men*. The majority of male participants believed that men had essentially no role to play in the raising of children below the age of 5, beyond providing sufficient income and adequate shelter for their families. One man commented, ‘From age one to five the child is not conscious, you can't communicate with him. You can only can take care of the child so he won't hurt himself.’ Another man suggested that ‘From zero to four there is nothing to talk about.’ Put simply, the early childhood content we had added was not perceived as relevant by the majority of men in the intervention.

In a related vein, on a new parenting questionnaire developed for this project that assesses parental warmth and sensitivity, harsh discipline, and understanding of child development, roughly 50% of women and men demonstrated several critical misconceptions about early childhood development. For example, about half the participants, *after completing the CSI*, still believed that the greatest period of brain development occurs after the age of 5, and that fathers have little impact on the psychosocial and cognitive development of their young children.

*Other points raised in the focus groups*. Several women requested more ‘quality time’ activities they could engage in with very young children, and to have these available in recorded form like the relaxation exercises. They also asked for a written manual with all of the parenting methods and activities learned in the sessions.

*Revisions to the CSI based on focus group and questionnaire data*. Based on data from the focus groups and the parenting questionnaire, we made several revisions to the intervention.

- Rather than introduce the first stress management exercise in Session one, we ask participants to share their own ways of coping with stress. Their home practice is to use one or more of the group-generated methods at least three times prior to the next session. We felt that men were asking for a recognition of their own capacity to cope with stress, and that their receptivity to learning new methods would be greater if we began with a recognition of their own coping strategies.
- A new activity was added to help participants understand the nature of the stress response, as well as the impact of prolonged stress on the body and mind. Scientific evidence regarding the benefits of countering stress with relaxation techniques was added, in easy to understand language, as were quotes from previous CSI participants who had practiced the exercises and found them to be helpful.
- We added a video activity showing men interacting playfully with their young children, and coupled this with a brief summary of findings regarding the powerful influence that fathers have on the psychosocial and cognitive development of their children. This new material was designed to address the misperception that men have no meaningful role to play with young children beyond providing for their material wellbeing. The use of video clips was intended to model and normalize the idea of fathers interacting in a nurturing way with young children.
- We added an interactive video-based activity focused on the power of early experience on young children's behavioral and emotional development. This activity was designed to help parents understand the pivotal role of early experience in child development, and the relationship between a nurturing environment and healthy brain development.
- We also added several quality time activities specifically for use with infants, toddlers, and young children; created a *Caregiver Manual*, which includes all stress management and parenting methods covered in the intervention; and added a movement-based stress management activity, ‘Peaceful Walking’, similar to the mindful walking commonly practiced in mindfulness trainings. Several participants had expressed difficulty sitting or lying still in order to do the stress management exercises, or said that they could not find a quiet place in their homes to sit quietly. Peaceful Walking was meant to be an alternative in such situations.

### Phase 3: implementation and evaluation of the revised CSI in a pilot randomized controlled trial

Following these revisions, we then conducted a pilot randomized controlled trial (RCT) of the CSI in greater Tripoli (Miller, Koppenol-Gonzalez *et al*., 2020). In this iteration, we aimed to recruit 72 families, in which *both* parents agreed to participate, a change from previous iterations in which only one parent from each family had been recruited. Having established that we could successfully recruit and retain men in the intervention, we now wished to see whether we could consistently recruit families in which both parents agreed to participate in the CSI.

Seventy-two families with both parents were successfully recruited (as well as seven families with just one participating parent). The mean age of participants was 36.6, with a range of 19–65. Mean number of children per household was 3.4. Focus groups were conducted with all CSI groups within 2 weeks following the last session. In total, 81% of participants attended the focus groups (34 women and 30 men). Focus group data from the pilot study were again coded and analyzed using NVivo v.12, and followed the same process described earlier.

#### Key findings

Both the quantitative and qualitative data suggest that the revisions made to the CSI satisfactorily addressed the concerns raised following the practice cycle. In total, 95% of the women and 86% of the men completed at least seven of the nine sessions (Miller, Koppenol-Gonzalez *et al*., 2020). Two women and five men dropped out of the intervention, with dropouts due to illness (four), conflicts with work (two), and an undetermined reason (one).

In contrast to the practice cycle, men in this iteration of the CSI were enthusiastic about the stress management exercises, citing as their favorites Peaceful Walking and Counting the Breath (a simple breath-focused activity aimed at quickly lowering arousal). Like their wives, men linked their use of the relaxation exercises to a greater sense of calm and wellbeing, and to a greater capacity to engage calmly and warmly with their children and spouses.

‘I used to enter the house angry and frowning. As soon as I entered, the kids would go into their room to avoid interacting with me. Now I enter smiling and calm, and the kids are now eager to interact with me. I also ask my wife to prepare dinner in a polite manner. The home atmosphere shifted and is now very beautiful and soothing.’

‘I used to get angry and break furniture at home very fast. This has completely changed after the program. Now, when I feel angry, I go out for a walk. Moreover, I now spend time with my children, play with them and take them for a ride. I was a different person before the program.’

Women confirmed the changes described by their husbands.

‘My husband was very irritable and always on edge. He now spends time with the children, he has become more understanding, and his interaction with them increased. Before the program, he never communicated with them, he only yelled, which made them terrified of him.’

The new activities focused on child development and the influence of fathers on their children's psychosocial wellbeing led to a substantial increase in men's nurturing involvement with their children.

‘The best information we learned was the growth of children between zero and five years of age, and how to interact with them, how to play with them. I love playing with my child and pretending that I am losing so he feels proud of himself. He gets very happy, as if he is the president of the country.’

‘We realized the importance of playing with children, and most importantly *how* to play with them. I'm more openly engaging with my children, creating a light and warm family atmosphere. I'm enjoying my time with my kids.’

These changes in their husbands' parenting behavior were also observed by women:

‘Back in Syria, my husband used to play with the children. However, since the events hit, he became irritable, without a job for long periods, and stopped spending time with the children. He is changed now. I am surprised how he has changed after the sessions. He now plays with them, and a couple of days ago he asked them to go inside and change so that he could take them for a ride. I was astonished, as for the past two years, he never once suggested taking them out.’

As in the previous implementation, women were enthusiastic about the program as a whole, and about the stress management exercises in particular, which they again linked to improved wellbeing and an increase in positive parenting.

‘My stress level has decreased by 80%. Even when I am happy I do the relaxation techniques. I practice the Safe Place all the time, in addition to the Peaceful Walking; these really affect me. I usually am a very irritable and stressed person. When I practice the Safe Place, I imagine heaven; I feel relaxed, satisfied, I laugh and smile, and I don't feel irritated anymore.’

‘The entire atmosphere in our home has changed. You used to feel this tension where everybody was angry before, but now, I learned how to absorb my anger and deal with it. I don't get angry in front of my kids anymore. I don't yell in front of them. The other day, my son got me very angry, so I retreated and took a few deep breaths to be able to deal with the situation.’

Finally, participants affirmed the importance of having both parents participate in the intervention. Women and men both stated that if the men had not participated, they would likely have been less supportive of the changes their wives were trying to make at home, and the impact on the family would have been less.

‘My wife and I both had attended and taken the sessions, and we encouraged each other through them. If she were to be alone in this, I would not have let her do anything.’

‘This is the first time we see such a program where the effort was made to involve men. If I take all this new info and I go home and my husband is the same, unchanged, it will not help. Now he's taking the same sessions and I started to feel he is changed, and that he is feeling better.’

Feedback from participants from the pilot RCT implementation indicated that the revisions we had made to the CSI had adequately addressed the concerns identified following the practice cycle. Higher attendance and lower drop-out rates reflected the highly positive responses shared during the focus groups. As no new concerns or issues were raised during this round of participant feedback, we determined that the CSI was ready for evaluation in a fully powered RCT (Miller, Arnous *et al*., 2020).

## Discussion

Our aim in this paper was to illustrate the essential role of participant feedback in the iterative process of shaping and strengthening a psychosocial intervention for conflict-affected parents. Focus group data from CSI participants in Gaza allowed us to establish the cultural acceptability and perceived usefulness of the intervention there, as well as the feasibility of recruiting and retaining Gazan men in a parent-focused intervention. Following substantive revision prior to implementing the CSI in Lebanon, focus group data with participants in the revised intervention enabled us to identify gaps in the curriculum as well as problematic elements of the intervention that needed revision. By listening and responding to the concerns of participants in the practice cycle, we were able to modify the CSI in ways that significantly enhanced the acceptability and perceived usefulness of the intervention. Other sources of data, such as participant responses on a newly developed parenting questionnaire, and expert consultation in the key content domains of the CSI, also played critical roles in helping us revise and strengthen the intervention.

### Preliminary support for the model underlying the CSI

Three waves of focus group data provided provisional support for the model underlying the CSI. Our findings suggest that under conditions of lower arousal, parents may be better able to make use of pre-existing as well as newly acquired parenting knowledge and skills. This calls into question the adequacy of efforts to strengthen parenting in settings of adversity that prioritize the acquisition of knowledge and skills, without attending substantively to parents' own wellbeing and the impact of stress and distress on their parenting behavior.

### The successful recruitment and retention of men

Panter-Brick *et al*. (2014*a*) have highlighted the paucity of parent-focused interventions that include fathers. After reviewing findings that underscore the powerful role that fathers have on all aspects of their children's psychological and psychosocial development, they caution that men's exclusion from parent-focused may significantly limit and even undermine intervention effects. This is because men may not understand or support the changes their partners are trying to enact, and may continue to engage in problematic parenting behaviors themselves (e.g. harsh parenting).

Before attempting to recruit men in the CSI, we first sought to understand the barriers that kept them from participating in parent-focused interventions. In consultation with our partner organizations in Gaza and Lebanon, and informed by the literature on parenting interventions, we identified three key obstacles: (1) a perception among men that parent-focused interventions are not relevant to them because of cultural norms proscribing their secondary role as caregivers of children; (2) an expectation among the staff of humanitarian organizations that men would not want to participate, and a decision to therefore allocate limited resources to recruiting and retaining women; and (3) conflicts arising from scheduling intervention and assessment sessions during times when men commonly engage in income-generating activities.

To address these concerns, we trained our recruitment staff in the theory and methods of the CSI. We also emphasized the importance of men as caregivers, and the positive role that men's participation could have on the success of the CSI. Second, we crafted a recruitment message emphasizing the focus on stress management, as well as raising children in difficult circumstances. Finally, we scheduled assessment and intervention sessions during evening and weekend hours in order to minimize conflicts with income-generating opportunities.

Thus far, these strategies have been successful. Most recently, we successfully recruited our target of 240 families in which both parents are participating in a fully powered RCT of the CSI (Miller, Arnous *et al*., 2020).

Finally, although we developed the CSI for families affected by armed conflict and forced migration, the emphasis on parental stress and psychosocial wellbeing suggests that it may be adaptable for use with families living in other high-stress environments where parenting may be adversely impacted by exposure to chronic adversity.

### Limitations

The positive findings on uptake and feasibility we have presented are primarily based on self-report in focus groups, among participants who certainly knew after completing the intervention what the aims of the CSI were. This raises the possibility that social desirability may have influenced their responses. However, we note that women often described changes in their husbands that matched the husbands' own self-reports; this also worked the other way, with men describing changes in their wives that corresponded closely to the women's self-reports. Our qualitative findings are also consistent with the quantitative data from the pilot RCT. Although intentionally underpowered, we found statistically significant improvement with medium or greater effect sizes on all study outcomes in the CSI arm, and no change on any outcome in the waitlist control group, lending further support to our focus group data (Miller, Koppenol-Gonzalez *et al*., 2020). Outcomes included caregiver wellbeing, stress, distress, stress management ability, parenting, and child psychosocial wellbeing.

The benefit of holding CSI sessions during evening and weekend hours that did not conflict with income generation opportunities may be somewhat offset by the challenge of finding staff willing to work during such unconventional hours. This has not been a problem for us thus far; however, this may be a challenge when it comes to scaling the CSI beyond the research phase.

## Footnotes

1 We use the term *parents* in this paper to refer all primary caregivers of children.
